# Prevalence of benefit finding and posttraumatic growth in long-term cancer survivors: results from a multi-regional population-based survey in Germany

**DOI:** 10.1038/s41416-021-01473-z

**Published:** 2021-07-02

**Authors:** Zhunzhun Liu, Melissa S. Y. Thong, Daniela Doege, Lena Koch-Gallenkamp, Heike Bertram, Andrea Eberle, Bernd Holleczek, Annika Waldmann, Sylke Ruth Zeissig, Ron Pritzkuleit, Hermann Brenner, Volker Arndt

**Affiliations:** 1 Unit of Cancer Survivorship, Division of Clinical Epidemiology and Aging Research, German Cancer Research Center (DKFZ), Heidelberg, Germany; 2 Medical Faculty of Heidelberg, University of Heidelberg, Heidelberg, Germany; 3 Division of Clinical Epidemiology and Aging Research, DKFZ, Heidelberg, Germany; 4 Cancer Registry of North Rhine-Westphalia, Bochum, Germany; 5 Bremen Cancer Registry, Leibniz Institute for Prevention Research and Epidemiology - BIPS, Bremen, Germany; 6 Saarland Cancer Registry, Saarbrücken, Germany; 7 Hamburg Cancer Registry, Hamburg, Germany; 8 Institute of Social Medicine and Epidemiology, University of Lübeck, Lübeck, Germany; 9 Cancer Registry of Rhineland-Palatinate, Mainz, Germany; 10 Cancer Registry of Schleswig-Holstein, Lübeck, Germany; 11 Division of Preventive Oncology, DKFZ and National Center for Tumor Diseases (NCT), Heidelberg, Germany; 12 German Cancer Consortium (DKTK), DKFZ, Heidelberg, Germany

**Keywords:** Epidemiology, Cancer epidemiology

## Abstract

**Background:**

Cancer studies reported mixed results on benefit finding (BF) and posttraumatic growth (PTG) prevalence and few were focused on long-term survivors.

**Methods:**

BF and PTG were assessed in a multi-regional population-based study in Germany with 6952 breast, colorectal and prostate cancer survivors, using the Benefit Finding Scale and Posttraumatic Growth Inventory. We calculated the age-adjusted prevalence, stratified by demographical and clinical characteristics.

**Results:**

Overall, 66.0% of cancer survivors indicated moderate-to-high BF, and 20.5% moderate-to-high PTG. Age-adjusted prevalence of BF and PTG differed according to cancer type (breast > colorectal > prostate) and sex (female > male). BF and PTG prevalence were higher in younger than in older respondents; the age-adjusted prevalence was higher in respondents who survived more years after diagnosis. The strength and direction of associations of age-adjusted prevalence with cancer stage, disease recurrence, and time since diagnosis varied according to cancer type and sex.

**Conclusions:**

A substantial proportion of long-term cancer survivors reported moderate-to-high BF and PTG. However, the prevalence was lower in older and male cancer survivors, and during the earlier years after cancer diagnosis. Further longitudinal studies on PTG and BF in cancer survivors are warranted to address heterogeneity in survivors’ experience after cancer diagnosis.

## Background

Cancer, as a life-threatening illness, has been recognised as a traumatic event [1]. However, over half of cancer survivors report at least one beneficial change or personal growth in cancer experience [2]. Derived from such positive perceptions, benefit finding (BF) [3] and posttraumatic growth (PTG) [4] have been conceptualised and widely researched. Both terms are often used synonymously in publications, but they differ. BF is a form of cognitive adaptation to adversity in which survivors positively evaluate their circumstance [3, 5]. PTG is defined as the positive changes experienced from a traumatic event [4]. BF assesses the broader and less specific positive changes compared to PTG, and the adversity is not necessary to be traumatic [6]. The experience of BF can start immediately after cancer diagnosis, while that of PTG can take a longer time to initiate due to essential processes (e.g. self-disclosure and rumination) that need to be first worked through [4]. For decades, researchers have found that cancer survivors with more BF and PTG reported higher health-related quality of life [7, 8]. Hence, clinicians recognise the importance of BF and PTG in cancer survivors [9, 10].

A challenging/traumatic event like cancer is crucial to trigger BF or PTG [4, 5]. Cancer trajectories vary depending on cancer type, stage, treatment and care protocols. Individuals’ approaches to post-diagnosis appraisal and coping (e.g. gain a sense of mastery) and the perception of BF and PTG in cancer experience vary as well [11]. Reported prevalence rates of BF and PTG range from 10% for PTG [12] to 99% for BF [13] in single-type cancer samples. Age and sex could affect feelings about cancer experience. A meta-analysis [14] showed that survivors of different trauma younger than 60 years were more likely than older survivors to report moderate-to-high PTG. On the other hand, older females have been considered to be cognitively more mature to comprehend the meaning of a cancer diagnosis and to experience personal growth than younger females [15]. Females are considered to be more emotionally sensitive to perceive the positive changes/growth from cancer experience than males [16]. In addition, research suggests that time is associated with the development of PTG and BF. However, the results are conflicting. On the one hand, studies suggested that time is needed [13, 17], and on the other hand, the recall of traumatic experiences reduces with increasing time since diagnosis [16]. Results from systematic reviews regarding the factors associated with prevalence of BF and PTG among cancer survivors are inconsistent [15, 18, 19]. Moreover, few studies focused on long-term (≥5 years post-diagnosis) cancer survivors [14].

Due to the increasing prevalence of cancer, it is crucial to better understand the outcomes of cancer-related stress and coping, such as BF and PTG. Most of the pertinent studies on the prevalence of BF and PTG have focused on heterogeneous cancer types and had mixed results [14, 20]. There is a lack of studies reporting the prevalence stratified by cancer type and further potential covariates. To fill these gaps, this study aims to describe age-specific and age-adjusted prevalence of BF and PTG in long-term cancer survivors according to cancer type, sex, cancer stage, experience of recurrence during follow-up, and time since cancer diagnosis. The findings might be useful for clinicians to understand and support cancer survivors’ recall of positive feelings from cancer experience in the long run.

## Methods

### Study participants

The study population came from the CAESAR study (Cancer survivorship—a multi-regional population-based study) which was conducted by the German Cancer Research Center (Deutsches Krebsforschungszentrum, DKFZ) in cooperation with population-based cancer registries in Germany. Participants in this study were registered in one of six population-based cancer registries in Germany (including the federal states of Schleswig-Holstein, Hamburg, Bremen, North Rhine-Westphalia (administrative district of Münster), Rhineland-Palatinate, and Saarland) [21].

Details of the recruitment of study participants including data collection have been described elsewhere [21]. In brief, the study included 20–75 years old (age at diagnosis) breast, prostate and colorectal cancer survivors diagnosed during 1994–2004. Data collection was conducted from August 2009 until April 2011 by postal questionnaires [22]. Of the 15,674 cancer survivors eligible for the study, 8631 did not participate, 91 completed a short questionnaire without BF or PTG items. Of the 6952 survivors who completed the long questionnaire (response rate: 44%), 3045 were breast cancer survivors, 1504 were colorectal cancer survivors (627 females, 877 males), and 2403 were prostate cancer survivors.

### Outcomes and measurements

#### Demographics and clinical data

The questionnaire used in CAESAR study included questions regarding demographic and clinical factors. Clinical data such as cancer type, cancer stage, year of birth, and year of diagnosis were provided by the cancer registries. Disease recurrence was self-reported and is defined as any recurrence, metastases or new cancer after index cancer. Respondents’ age was calculated by deducting year of birth from year of survey; years since diagnosis equate to the year of survey minus the year of diagnosis.

#### Benefit finding

Benefit finding (BF) was measured by the German short form of the Benefit Finding Scale (BFS) [23]. The original [24] and German version [23] are valid and reliable. The 10-item BFS is scored on five-point Likert scales ranging from 1 (not at all) to 5 (extremely). Items are classed into four subscales (acceptance/sensitive to others/improving coping/ new purpose of life).

#### Posttraumatic growth

Posttraumatic growth (PTG) was assessed by three subscales (appreciation of life/spiritual change/new possibilities) of the German version of the Posttraumatic Growth Inventory (PTGI) [25]. Two other scales, ‘personal strength’ and ‘relationship to others’ were not included [13], given their overlaps with BFS and also to limit the length of the total questionnaire to reduce participant burden [6]. The original [26] and German version [25] are valid and reliable. The 10-item PTGI in this study uses six-point Likert scales ranging from 0 (I did not experience this change as a result of my cancer) to 5 (I experienced this change to a very great degree as a result of my cancer).

#### Intensity of BF and PTG

The intensity levels of BF and PTG were determined by the mean item score. The mean item score was calculated as the mean of non-missing items if at least half of the items had been completed, which was then dichotomised using a cut-off of 3 (indicated as ‘moderate’ according to a previous report [27]) into ‘none-to-low’ and ‘moderate-to-high’ (Supplementary Table 1).

### Statistical analysis

Multiple imputation with 25 repetitions was employed to handle missing values by using the Markov chain Monte Carlo method to reduce possible bias and increase precision of the replaced values [28]. Before analysing prevalence, we examined the reliability and validity [29] within each individual instrument (Supplementary Table 2-1) and across instruments (Supplementary Table 2-2) of the BFS and PTGI used in this study. Within instruments, BFS and PTGI demonstrated acceptable internal consistency (Cronbach’s α > 0.70) and validity (Supplementary Table 2-1). BFS and PTG were highly correlated (odds ratio = 23.3, co-prevalence of BF and PTG are presented in Supplementary Table 3) but the reliability and validity of the two instruments differed. Although there were overlaps between BFS and PTGI (especially for the subscale “new purpose of life”, which showed high convergent and low discriminant validity with PTGI, see Supplementary Table 2-2), differences existed in the subscales between BFS and PTGI. For BFS, the acceptance subscale showed low convergent and high discriminant validity with PTGI; and for PTGI, the spiritual change subscale showed low convergent and high discriminant validity with BFS.

In the description of characteristics of the total study population, and by cancer type and sex, respondents’ were grouped according to age at survey: <60 years, 60–69 years, 70–79 years, or 80–89 years; and to their time since diagnosis: long-term cancer survivors (LTCS; 5–9 years post-diagnosis) or very long-term cancer survivors (VLTCS; 10 or more years post-diagnosis). Age-adjusted prevalence of moderate-to-high BF and PTG by cancer type and sex were calculated according to the direct method using the age-specific proportions from the total sample described previously as standard population weights. Distributions of moderate-to-high BF and PTG prevalence according to age groups in cancer survivors overall and by cancer type and sex were also estimated. Detailed age-adjusted prevalence of moderate-to-high BF and PTG by characteristics of the study population on overall scale level were calculated accordingly, stratified by cancer type and sex. Chi^2^-tests were used to evaluate subgroup differences. A two-tailed *P*-value <0.05 was considered statistically significant.

## Results

### Sample characteristics

The imputed data was comparable to original data (Table 1). From the imputed data, mean respondents’ age was 69.1 ± 8.9 years, and mean time since diagnosis was 8.0 ± 2.2 years. Nearly three quarters of the survivors were diagnosed with cancer at UICC stage I/II, and 12.9% had experienced cancer recurrence.

**Table 1:** Characteristics of study population overall and by cancer type and sex.

	Overall survivors		Breast (female)		Colorectal (female)		Colorectal (male)		Prostate (male)	
	No. (%col)	MI %col	No. (%col)	MI %col	No. (%col)	MI %col	No. (%col)	MI %col	No. (%col)	MI %col
Total	6952 (100)	–	3045 (100)	–	627 (100)	–	877 (100)	–	2403 (100)	–
*Age at survey*										
<60 years	1041 (15.0)	15.0	822 (27.0)	27.0	82 (13.1)	13.1	86 (9.8)	9.8	51 (2.1)	2.1
60–69 years	2071 (29.8)	29.8	1098 (36.1)	36.1	164 (26.2)	26.2	248 (28.3)	28.3	561 (23.3)	23.4
70–79 years	3165 (45.5)	45.5	973 (32.0)	32.0	286 (45.6)	45.6	438 (49.9)	49.9	1468 (61.1)	61.1
80–89 years	674 (9.7)	9.7	152 (5.0)	5.0	95 (15.2)	15.2	105 (12.0)	12.0	322 (13.4)	13.4
Missing	1 (0.0)	–	–	–	–	–	–	–	1 (0.0)	–
*Years since diagnosis*										
5–9 years	5419 (78.0)	78.4	2336 (76.7)	76.9	395 (63.0)	64.3	621 (70.8)	71.3	2067 (86.0)	86.7
10–16 years	1488 (21.4)	21.6	702 (23.1)	23.1	221 (35.2)	35.7	250 (28.5)	28.7	315 (13.1)	13.3
Missing	45 (0.6)	–	7 (0.2)	–	11 (1.8)	–	6 (0.7)	–	21 (0.9)	–
*UICC stage at diagnosis*										
I/II	4320 (62.1)	73.9	2546 (83.6)	90.3	314 (50.1)	64.4	482 (55.0)	67.0	978 (40.7)	58.2
III/IV	1331 (19.2)	26.1	264 (8.7)	9.7	186 (29.7)	35.6	250 (28.5)	33.0	631 (26.3)	41.8
Missing	1301 (18.7)	–	235 (7.7)	–	127 (20.3)	–	145 (16.5)	–	794 (33.0)	–
*Recurrence/metastases*										
Yes	853 (12.3)	12.9	321 (10.5)	11.1	64 (10.2)	10.7	126 (14.4)	15.0	342 (14.2)	15.0
No	5793 (83.3)	87.1	2593 (85.2)	88.9	541 (86.3)	89.3	715 (81.5)	85.0	1944 (80.9)	85.0
Missing	306 (4.4)	–	131 (4.3)	–	22 (3.5)	–	36 (4.1)	–	117 (4.9)	–

*MI* multiple imputation, *%col* column-percent.

### Age-adjusted prevalence of moderate-to-high BF and PTG by cancer type and sex

**Fig. 1:**
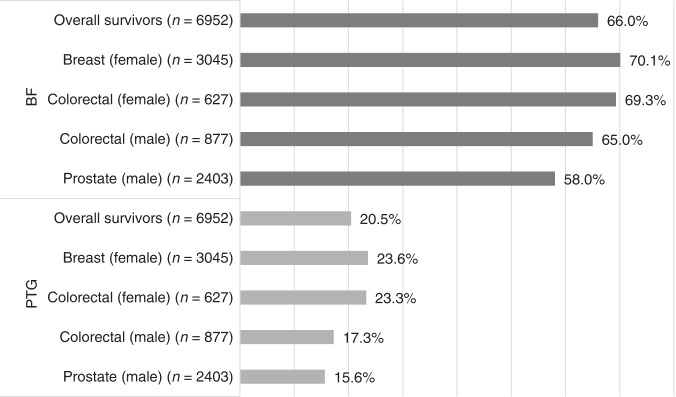
Age-adjusted prevalence of moderate-to-high benefit finding (BF) and posttraumatic growth (PTG) in cancer survivors overall and by cancer type/sex.

Overall, 66.0% of all survivors indicated moderate-to-high BF (95%CI: 65.8%–66.2%) and 20.5% moderate-to-high PTG (95%CI: 20.3%–20.7%) (Fig. 1). Similar patterns of moderate-to-high BF and PTG were observed across cancer type and sex after adjusting for respondents’ age. The age-adjusted prevalence of moderate-to-high BF, as well as PTG, was significantly different between cancer types and sex (*P* < 0.0001). Stratifying all survivors by sex, the age-adjusted prevalence was statistically significantly higher in female than in male cancer survivors, both for moderate-to-high BF (69.8% females: 61.0% males, *P* < 0.0001) and moderate-to-high PTG (23.5% females: 16.4% males, *P* < 0.0001).

### Age-specific and age-adjusted prevalence of moderate-to-high BF and PTG by sample characteristics, stratified by cancer type and sex

**Fig. 2:**
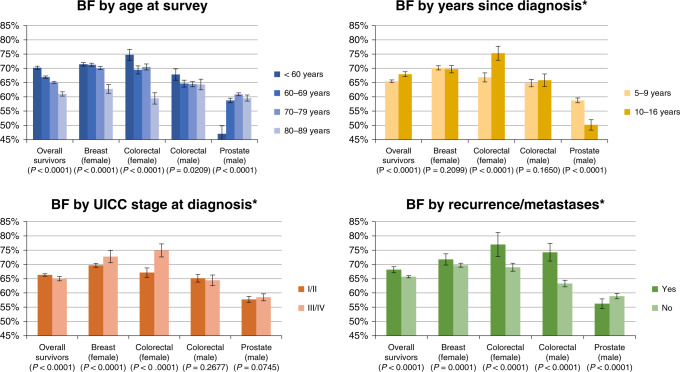
Prevalence of moderate-to-high benefit finding (BF) by age, years since diagnosis, cancer stage, and disease status, overall and according to cancer type and sex. Note: *P*-values were calculated from Chi^2^-test of subgroup differences of independent variables. *Age-adjusted prevalence.

**Fig. 3:**
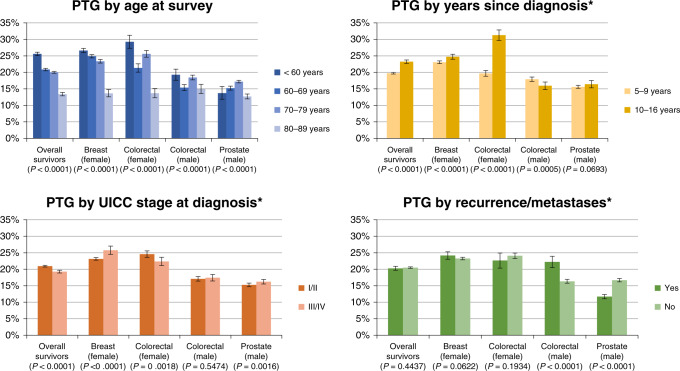
Prevalence of moderate-to-high posttraumatic growth (PTG) by age, years since diagnosis, cancer stage, and disease status, overall and according to cancer type and sex. Note: *P*-values were calculated from Chi^2^-test of subgroup difference of independent variable. *Age-adjusted prevalence.

Pattern of age-specific prevalence: a significant trend by age was noted in which older survivors reported lower prevalence of moderate-to-high BF and PTG. When stratified by cancer type, the same pattern was observed in breast cancer survivors (Figs. 2 and 3). Comparing all subgroups stratified by cancer type and sex, the highest prevalence of moderate-to-high BF and PTG was observed in the younger than 60 years old female colorectal cancer survivors group (Figs. 2 and 3).

Age-adjusted prevalence by other characteristics: Overall, survivors of stage III/IV cancer reported lower prevalence of moderate-to-high BF (*P* < 0.0001) and PTG (*P* < 0.0001) as compared to stage I/II survivors (Figs. 2 and 3). When stratified by cancer type and sex, reverse patterns and general heterogeneity were found. Statistically significant differences were detected when stratified by cancer type and sex, except for prostate cancer survivors on the prevalence of moderate-to-high BF (Fig. 2), and male colorectal cancer survivors on the prevalence of moderate-to-high BF and PTG (Figs. 2 and 3).

In general, survivors having had an experience of cancer recurrence indicated less BF than survivors without recurrence or metastasis (*P* < 0.0001). This pattern was found in all tumour groups but reversely in prostate cancer survivors (Fig. 2). For PTG, no significant differences in the prevalence of moderate-to-high PTG were found between survivors with and without recurrence of disease in the overall survivors, breast cancer survivors, and female colorectal cancer survivors, but contradicting patterns were found for male colorectal cancer and prostate cancer (Fig. 3).

Regarding time since diagnosis, LTCS showed a significantly lower age-adjusted prevalence of moderate-to-high BF (Fig. 2) and moderate-to-high PTG than VLTCS (Fig. 3). However, this pattern varied according to cancer type and sex. Time since diagnosis was inversely associated with the prevalence of moderate-to-high BF in prostate cancer survivors (*P* < 0.0001) but positively associated with the prevalence of moderate-to-high BF in female colorectal cancer survivors. Time since diagnosis was positively associated with the prevalence of moderate-to-high PTG in breast and female colorectal cancer survivors but inversely associated in male colorectal cancer survivors (*P* = 0.0005).

## Discussion

Based on data from a multi-regional population-based study, our study contributes detailed information on the prevalence of BF and PTG according to demographic and disease characteristics. When adjusted for respondents’ age, our results suggest that the prevalence of BF and PTG differed by cancer type (breast > colorectal > prostate) and sex (female > male). Generally, the prevalence of moderate-to-high BF and PTG is lower in older cancer survivors compared to younger age groups; the age-adjusted prevalence of moderate-to-high BF and PTG was higher in VLTCS than in LTCS. Cancer stage, disease recurrence and time since diagnosis were also associated with the prevalence of BF and PTG, although the strength and direction of the association varied according to cancer type and sex. To our knowledge, our study is the largest one to address the role of time since diagnosis on the prevalence of moderate-to-high BF and PTG in cancer survivors 5–16 years after diagnosis, with sufficient power to describe the prevalence by cancer type and sex in detail.

Age, in general, had an inverse relationship with PTG and BF, which is comparable to previous studies [14, 30]. The results suggest that being confronted with a cancer diagnosis at a younger age is more threatening than at an older age, thereby inducing the greater potential for initiation of the BF and PTG process. One further explanation is that age can be associated with life cycle events, e.g. retirement or bereavement, experienced by cancer survivors beyond their cancer; aging may also correspond to a decline in health and function [30]. The greater the number and severity of experienced life and health-related events, the higher the traumatic stress level an individual may be able to endure. Stanko and colleagues [31] suggested another explanation that the older (age > 65) lost hope in having positive changes occur after a trauma. While lower life expectations would reduce the discrepancy between one’s original beliefs (towards the world, self, and self-in-world) and trauma meaning; then less distress would be perceived and less BF and PTG would be experienced [32]. Most respondents in this study were older than 60 years when they were diagnosed with cancer. Therefore, the prevalence of moderate-to-high PTG in this study was lower compared to other studies with younger samples [14, 33].

In our study, the prevalence of moderate-to-high PTG ranged between 16 and 24%. These rates are lower compared to previous studies in a recent review [14]. It is possible that these differences with previous studies might be related to cultural differences, e.g. 25% of breast cancer survivors in our sample reported moderate-to-high PTG. This is in contrast to 60–73% in a Chinese sample, 66% in a Polish sample, and 56% in a Portuguese sample [14]. However, due to the heterogeneity of the sample characteristics and different cut-off values used in previous studies, it is difficult to directly compare the prevalence among the studies using samples from other cultures. Furthermore, our sample is older than those from previous studies [14], which might also be related to the low prevalence. Future studies could address potential cross-cultural differences of BF and PTG.

Following adjustment by respondents’ age, our study found prevalence differences in BF and in PTG according to sex and cancer type. Reflecting the sex distribution of the cancer types, the highest prevalence rates of BF and PTG were noted in breast cancer survivors, and the lowest prevalence in prostate cancer survivors in this study. The sex difference (higher prevalence was found in females than in males) also existed in colorectal cancer survivors. Life cycle events, physical changes, and developmental maturity associated with aging could also differ by sex. Females experience more stressful life events with physical changes, like pregnancy, giving birth or (peri)menopause [34, 35]. They may also be more sensitive to perceive a situation as threatening and stressful, and experience more psychological and biological responses to trauma [36]. Existing meta-analyses regarding BF and PTG indicated that females reported more BF and PTG than males [2, 30], and that this trend increases with increasing mean age of the sample.

Cancer stage may also contribute to differences in the prevalence of PTG and BF. The lower chance of cancer-free survival associated with more advanced cancer stage could increase the sense of hopelessness and perception of illness in a negative way [37]. As a result, cancer survivors with more advanced cancer stage might have reported lower BF and PTG. This association of cancer stage with BF and PTG is confirmed in this study, even when stratified by sex or cancer types, which is comparable to previous studies that only focused on a single cancer (e.g. breast cancer [38], colorectal cancer [13]).

We found that long-term cancer survivors, with or without recurrence, reported comparable prevalence of PTG, but those with cancer recurrence/metastasis generally had a higher chance to perceive BF. Previous studies also reported that long-term survivors with adolescent cancer who had recurrence/metastasis did not significantly differ in terms of their amount of PTG from those who did not experience such an episode [13, 39]. However, these results are based on cross-sectional data. Longitudinal research design could better describe the trajectory of PTG before and after recurrence. Cancer recurrence/metastasis is also a challenging or traumatic event. As PTG needs time to get through the initiation processes (e.g. self-disclosure and rumination) [4], and BF could be experienced soon after the event, time since recurrence could be the potential confounder that influence the recurrence and PTG/BF. It is also possible that personality traits (e.g. optimism, positive reframing) could play a role [2, 18]. However, this study did not include personality traits. Further studies could investigate the associations between perceived illness severity and personality traits on BF after disease recurrence in long-term cancer survivors.

While previous research only included short-term survivors (within 5 years) [13, 40], we included long-term cancer survivors (5–16 years post-diagnosis) in this study and generally found that longer time since diagnosis was associated with more moderate-to-high BF and PTG in this study. As the recall of traumatic experience could be reduced and trauma-related stress could decline with time, the relationship between PTG and the passage of time since diagnosis may be more positive and stronger in the immediate years following diagnosis and treatment than after several years of survivorship [41]. Our results support the assumption that time since diagnosis has positive relationships with BF and with PTG, and these relationships may last for more than 5 years. However, the cross-sectional samples in this study were 5–16 years past diagnosis, which suggests selection bias (healthy survivor bias) could exist. Those who survive from cancer for over 5 years may be more grateful and report higher BF/PTG. Taken together, further evidence from longitudinal studies with repeated BF/PTG assessments is needed to conclude on the actual association between time since diagnosis and BF/PTG.

### Limitations

Although our reports were from a population-based study, which allowed the detailed stratification according to several factors, several limitations still need to be noted. Firstly, we acknowledge that potential response bias could exist because of an over-selection of ‘fitter’ survivors who were able to complete the questionnaire or were more interested/motivated to give feedback. Secondly, there is a limited generalisation with regard to sex differences as we only looked at selected cancer types. Nevertheless, these three cancers are the most common cancers for males and females (apart from lung cancer, for which the proportion of long-term survivors is very low). Another factor related to cancer type is that the age distribution of samples differed between the cancers. To address this age difference, we reported age-adjusted prevalence when we stratified the groups by cancer type and sex. Thirdly, we did not use full instruments because we wanted to reduce participant burden by excluding overlapping subscales. However, the mean ratings of two excluded subscales of PTGI reported in other studies [42, 43] were almost the same as the subscales included in our study. As such, the result that a higher prevalence of BF compared to PTG was reliable even though we only used three subscales of PTGI. Fourthly, there are no universal cut-offs for BFS and PTGI. We chose a published criterion previously used in a German sample as this minimises the potential bias of using a shortened version of the BFS and PTGI. Our results could reflect the difference between the two concepts; BF describes broader and less specific positive changes compared to PTG. However, the difference in prevalence between BF and PTG is intriguing and should be investigated in future studies [13].

Future studies on the prevalence of BF/PTG in cancer survivors need to address the abovementioned limitations. For example, they could use full instruments and include more types of cancers to verify the results. Future prospective studies should ideally assess baseline BF/PTG before the onset of cancer or at least at diagnosis and longitudinal studies with repeated BF/PTG assessments could verify the association between time since diagnosis and BF/PTG. Additional studies are also needed to test cognitive perception of illness severity in processing of BF and PTG.

## Conclusion

We found that moderate-to-high BF and PTG are common in cancer survivors 5–16 years after diagnosis and that the prevalence varies with survivors’ age, sex, cancer type, stage of disease, recurrence/metastasis, and time since diagnosis. Our results highlight the heterogeneity in survivors’ experience after cancer diagnosis. Prevalence of BF and PTG was lower in older and male cancer survivors, during the earlier years after diagnosis and in survivors with more advanced cancer diagnosis. Further longitudinal studies on PTG and BF in cancer survivors are warranted to address heterogeneity in survivors’ experience after cancer diagnosis. Better identification of vulnerable survivors, e.g. taking demographic and clinical factors into consideration, could assist in tailoring interventions appropriate to their needs.

## Supplementary information

supplemental material

## Data Availability

Not applicable.
