# Supplementary material for: Prevalence of benefit finding and posttraumatic growth in long-term cancer survivors: results from a multi-regional population-based survey in Germany
Source: Br J Cancer. 2021 Jul 2;125(6):877–83. doi: 10.1038/s41416-021-01473-z (PMC8437934; doi:10.1038/s41416-021-01473-z)
Supplement: Supplementary file 1 — supplemental material [file 41416_2021_1473_MOESM1_ESM.docx]

Supplementary Table 1. Intensity levels and dichotomization of BF/PTG and related groups of BF&PTG

| Scale | answers/item | score/item | Intensity levels/scale | Mean scale score/item | Dichotomizations of BF/PTG |
| --- | --- | --- | --- | --- | --- |
| BFS | Not at all | 1 | None BF | 1 | None-to-Low BF |
|  | A little | 2 | Low BF | 1 < BF < 3 |  |
|  | Moderately | 3 | Moderate BF | 3 | Moderate-to-High BF |
|  | Quite a bit | 4 | High BF | 3 < BF < 5 |  |
|  | Extremely | 5 | Extremely high BF | 5 |  |
| PTGI | Not at all | 0 | None PTG | 0 | None-to-Low PTG |
|  | To a very small degree | 1 | Low PTG | 0 < PTG < 3 |  |
|  | To a small degree | 2 |  |  |  |
|  | To a moderate degree | 3 | Moderate PTG | 3 | Moderate-to-High PTG |
|  | To a great degree | 4 | High PTG | 3 < PTG < 5 |  |
|  | To a very great degree | 5 | Very high PTG | 5 |  |

Supplementary Table 2-1. Reliability and validity of individual instrument

|  | No. of items | Mean ± SD | α | Convergent | | | | Discriminant | | St. Factor  Loading | AVE | Sqrt (AVE) | CR |
| --- | --- | --- | --- | --- | --- | --- | --- | --- | --- | --- | --- | --- | --- |
|  |  |  |  | RC _item-to-own scale_ | SS^0^ (%) | RC _scale-to-scale_ | SS^0^ (%) | RC _item-to-other scale_ | SS^1^ (%) |  |  |  |  |
| **PTGI** | 10 | 1.96±1.10 | 0.91 | 0.67-0.81^*^ | 10/10(100)^*^ |  |  |  |  |  | 0.66 | 0.81 | 0.95 |
| Spiritual change | 2 | 1.49±1.42 | 0.89 | 0.95^&^ | 2/2 (100) | 0.49-0.59 | 2/2 (100) | 0.42-0.59 | 4/4 (100) | 0.83-0.97 | 0.81 | 0.90 | 0.90 |
| Appreciation of life | 3 | 2.85±1.28 | 0.83 | 0.81-0.90 | 3/3 (100) | 0.49-0.70 | 2/2 (100) | 0.41-0.67 | 6/6 (100) | 0.65-0.87 | 0.64 | 0.80 | 0.84 |
| New possibilities | 5 | 1.64±1.16 | 0.89 | 0.80-0.86 | 5/5 (100) | 0.59-0.70 | 2/2 (100) | 0.46-0.67 | 10/10 (100) | 0.72-0.79 | 0.61 | 0.78 | 0.89 |
| **BFS** | 10 | 3.27±0.96 | 0.90 | 0.59-0.82^*^ | 10/10(100)^*^ | 0.68-0.91^**^ | 4/4 (100)^**^ |  |  |  | 0.56 | 0.75 | 0.93 |
| Acceptance | 2 | 3.55±1.08 | 0.83 | 0.92-0.93 | 2/2 (100) | 0.43-0.51 | 3/3 (100) | 0.37-0.51 | 6/6 (100) | 0.77-0.92 | 0.72 | 0.85 | 0.84 |
| Sensitive to others | 3 | 3.23±1.13 | 0.72 | 0.78-0.81 | 3/3 (100) | 0.43-0.71 | 3/3 (100) | 0.33-0.63 | 9/9 (100) | 0.62-0.73 | 0.45 | 0.67 | 0.71 |
| Improved coping | 3 | 3.19±1.12 | 0.81 | 0.81-0.87 | 3/3 (100) | 0.51-0.81 | 3/3 (100) | 0.40-0.79 | 9/9 (100) | 0.66-0.87 | 0.58 | 0.76 | 0.81 |
| New purpose of life | 2 | 3.15±1.20 | 0.70 | 0.87-0.88 | 2/2 (100) | 0.49-0.81 | 3/3 (100) | 0.40-0.79 | 6/6 (100) | 0.65-0.83 | 0.55 | 0.74 | 0.71 |

Note: Cronbach’s α was calculated as a measure of internal consistency. Validity was tested by defined convergent validity, discriminant validity and confirmatory factor analysis indicators, incl. St. Factor Loading, AVE, Sqrt (AVE) and CR.

Abbreviations: RC: Range of Correlation; SS: Scaling Success; St.: standardized; AVE: Average Variance Extracted; CR: Composite Reliability; Sqrt: Square root.

^&^0.9486-0.9491, ^*^ item-to-own instrument correlation, ^**^scale-to-own instrument correlation, subscales of PTGI used in this study was not included in this correlation analysis.

^0^ Scaling success of convergent is counted if the correlation coefficient of item-to scale or scale-to-instrument is higher than 0.4.

^1^ The correlation coefficient regarding the item-to-own scale is higher than item-to-other scale within the instrument.

Supplementary Table 2-2. Convergent and discriminant validity across instruments (correlation between PTGI & BFS = 0.70)

|  | Item level validity | | | Scale level validity | | | Two-factor-model | | | | One factor | | | |
| --- | --- | --- | --- | --- | --- | --- | --- | --- | --- | --- | --- | --- | --- | --- |
|  | RC _item-to-scale_ | Convergent | Discriminant | RC _scale-to-scale_ | Convergent | Discriminant | St. Factor  Loading | AVE | Sqrt (AVE) | CR | St. Factor  Loading | AVE | Sqrt (AVE) | CR |
|  |  | SS^0^ (%) | SS^2^ (%) |  | SS^0^ (%) | SS^3^ (%) |  |  |  |  |  |  |  |  |
| **PTGI** | 0.37-0.66^*^ | 9/10 (90)^*^ | 100/100 (100) ^*^ | 0.45-0.70^**^ | 3/3 (100)^**^ | 9/9 (100)^**^ |  |  |  |  |  |  |  |  |
| Spiritual change | 0.17-0.49 | *3/8 (38)* | 24/24 (100) | 0.21-0.47 | *2/4 (50)* | 8/8 (100) | *0.62* | 0.60 | 0.78 | 0.82 | *0.55* |  |  |  |
| Appreciation of life | 0.34-0.65 | 9/12 (75) | 26/36 (72) | 0.41-0.70 | 4/4(100) | 5/8 (63) | 0.85 |  |  |  | 0.78 |  |  |  |
| New possibilities | 0.22-0.60 | 14/20 (70) | 51/60 (85) | 0.32-0.65 | 3/4 (75) | 6/8 (75) | 0.84 |  |  |  | 0.74 |  |  |  |
| **BFS** | 0.31-0.69^*^ | 8/10 (80)^*^ | 92/100 (92) ^*^ | 0.37-0.71^**^ | 3/4 (75)^**^ | 14/16 (88)^**^ |  |  |  |  |  |  |  |  |
| Acceptance | 0.18-0.41 | *1/6 (17)* | 24/24 (100) | 0.21-0.42 | *1/3 (33)* | 9/9 (100) | *0.55* | 0.63 | 0.80 | 0.87 | *0.54* |  |  |  |
| Sensitive to others | 0.26-0.50 | 4/9 (44) | 29/36 (81) | 0.35-0.57 | 2/3 (67) | 7/9 (78) | 0.77 |  |  |  | 0.77 |  |  |  |
| Improved coping | 0.33-0.69 | 7/9 (78) | 27/36 (75) | 0.46-0.70 | 3/3 (100) | 7/9 (78) | 0.91 |  |  |  | 0.90 |  |  |  |
| New purpose of life | 0.40-0.65 | 6/6 (100) | 18/24 (75) | 0.47-0.65 | 3/3 (100) | 7/9 (78) | 0.89 |  |  |  | 0.88 |  |  |  |
| Total |  |  |  |  |  |  |  | **0.62** | **0.79** | **0.92** |  | **0.56** | **0.75** | **0.90** |
| Criteria |  | > 50% |  |  | > 50% |  | > 0.70 | > 0.50 |  | > 0.70 | > 0.70 | > 0.50 |  | > 0.70 |

Note: The italic results did not meet the criteria.

Abbreviations: RC: Range of correlation; SS: Scaling success; AVE: Average Variance Extracted; CR: Composite Reliability; Sqrt: Square root.

^*^ item-to-other instrument correlation, ^**^scale-to-other instrument correlation

^0^ Scaling success of convergent is counted if the correlation coefficient of item-to scale or scale-to-instrument is higher than 0.4.

^1^ The correlation coefficient regarding the item-to-own scale is higher than item-to-other scale within the instrument.

^2^ The scaling success is counted if the item-to-other instrumental scale coefficient is lower than the all item-to-own instrumental scale coefficients.

^3^The correlation coefficient regarding the scale-to-own instrument scales is higher than item-to-other instrument scales within the instrument.

Supplementary Table 3. Co-prevalence of BF and PTG

|  | | PTG | | |
| --- | --- | --- | --- | --- |
|  |  | Moderate to high | None to Low | Total |
| BF | Moderate to high | 18.3% | 46.1 % | 64.4% |
|  | None to Low | 0.6 % | 35.0 % | 35.6% |
|  | Total | 18.9% | 81.1% | 100.0% |
